# Morphine‐induced respiratory depression is independent of β‐arrestin2 signalling

**DOI:** 10.1111/bph.15004

**Published:** 2020-02-17

**Authors:** Andrea Kliewer, Alexander Gillis, Rob Hill, Frank Schmiedel, Chris Bailey, Eamonn Kelly, Graeme Henderson, Macdonald J. Christie, Stefan Schulz

**Affiliations:** ^1^ Institute of Pharmacology and Toxicology Jena University Hospital, Friedrich‐Schiller‐University Jena Germany; ^2^ Discipline of Pharmacology, School of Medical Sciences University of Sydney Sydney NSW Australia; ^3^ School of Physiology, Pharmacology and Neuroscience University of Bristol Bristol UK; ^4^ Department of Pharmacy and Pharmacology University of Bath Bath UK

## Abstract

**Background and Purpose:**

GPCRs can signal through both G proteins and β‐arrestin2. For the μ‐opioid receptor, early experimental evidence from a single study suggested that G protein signalling mediates analgesia, whereas β‐arrestin2 signalling mediates respiratory depression and constipation. Consequently, for more than a decade, much research effort has been focused on developing biased μ‐opioid agonists that preferentially target G protein signalling over β‐arrestin signalling, as it was believed that such drugs would be analgesics devoid of respiratory depressant activity. However, the prototypical compounds that have been developed based on this concept have so far failed in clinical and preclinical development.

**Experimental Approach:**

The present study was set up to re‐examine opioid‐induced respiratory depression in β‐arrestin2 knockout mice. To this end, a consortium was formed consisting of three different laboratories located in different countries to evaluate independently opioid‐induced respiratory depression.

**Key Results:**

Our consensus results unequivocally demonstrate that the prototypical μ‐opioid agonist morphine (3.75–100 mg·kg^−1^ s.c. or 3–30 mg·kg^−1^ i.p.) as well as the potent opioid fentanyl (0.05–0.35 mg·kg^−1^ s.c.) do indeed induce respiratory depression and constipation in β‐arrestin2 knockout mice in a dose‐dependent manner indistinguishable from that observed in wild‐type mice.

**Conclusion and Implications:**

Our findings do not support the original suggestion that β‐arrestin2 signalling plays a key role in opioid‐induced respiratory depression and call into question the concept of developing G protein‐biased μ‐opioid receptor agonists as a strategy for the development of safer opioid analgesic drugs.

**Abbreviation**
βarr2−/−: β‐arrestin2 knockout

**What is already known**

- Data suggests that μ‐opioid analgesia is via G protein; respiratory depression/constipation is via β‐arrestin2 signalling.
- However, compounds based on the concept of biased agonism failed in clinical and preclinical development.

**What this study adds**

- μ‐Opioid agonists morphine and fentanyl induce respiratory depression and constipation in β‐arrestin2 knockout mice.

**What is the clinical significance**

- These results question G protein‐biased agonists as a strategy for safer opioid analgesic drugs.

## 1 INTRODUCTION

Regulatory β‐arrestin proteins, downstream of GPCRs, are widely understood to mediate receptor desensitisation. Binding of β‐arrestins to phosphorylated GPCRs is also believed to trigger a second wave of G protein‐independent signalling (Reiter, Ahn, Shukla, & Lefkowitz, 2012). The discovery of ligands that preferentially stimulate G protein or β‐arrestin2 signalling has led to the concept of “biased agonism” or “functional selectivity.” However, for most GPCRs, it is still unclear which *in vivo* physiological responses are mediated by G protein signalling and which responses are mediated by β‐arrestin2 signalling. Nevertheless, there is currently a growing interest in the development of functionally selective GPCR ligands facilitated by hopes of producing more effective drugs with reduced side effects (Wacker, Stevens, & Roth, 2017). In the development of new opioid analgesic drugs, a key driving force was a study published in 2005 on β‐arrestin2 knockout mice that reported enhanced and prolonged analgesic effects after morphine application, presumably due to decreased receptor desensitisation, and reduced respiratory depressant and constipatory effects (Raehal, Walker, & Bohn, 2005). These results led to the novel concept that opioid analgesia is mediated via G protein signalling, while respiratory depression and constipation are mediated predominantly via β‐arrestin‐dependent signalling pathways. Despite much research effort devoted towards the development of novel G protein‐biased μ‐opioid agonists, this hypothesis has not been verified thus far. The first prototypical compound oliceridine (TRV130) recently completed phase 3 clinical studies but failed to demonstrate superiority to morphine in terms of respiratory safety burden in humans (Hertz, 2018). Another potentially G protein‐biased μ‐opioid agonist, PZM21, was discovered using a structure‐based approach (Manglik et al., 2016). Although initially reported to produce fewer side effects, a later study clearly demonstrated that PZM21 still produced pronounced respiratory depression in mice (Hill et al., 2018).

Moreover, the biased agonism concept was recently tested using a different approach. By generating knock‐in mice with a series of carboxyl‐terminal S/T to alanine mutations that are increasingly unable to recruit regulatory β‐arrestin proteins, a novel model was created that allows genetic dissection of signalling bias at the μ‐receptor level (Kliewer et al., 2019). As phosphorylation‐deficient receptors signal via G proteins for prolonged periods without apparent desensitisation but at the same time fail to recruit β‐arrestin proteins, these mutants can be viewed as G protein‐biased μ‐opioid receptors. Mice expressing phosphorylation‐deficient, G protein‐biased μ‐opioid receptors showed enhanced analgesia in response to morphine, but, contrary to prediction, they still exhibited respiratory depression and other opioid‐induced side effects such as constipation.

Given these contradictory results, we formed a consortium consisting of three different laboratories to re‐examine opioid‐induced respiratory depression in β‐arrestin2 knockout mice in a systematic manner.

## 2 METHODS

### 2.1 Jena, Germany

#### 2.1.1 Animals

β‐Arrestin2 knockout mice strain B6.129‐Arrb2^tm1Rjl^/J (βarr2−/−, RRID:IMSR_JAX:011130) were obtained from JAX™/Charles River Laboratories (DE). Mice were genotyped by PCR analysis of genomic tail‐biopsy DNA using the following primers: 5′‐GCTAAAGCGCATGCTCCAGA‐3′, 5′‐ACAGGGTCCACTTTGTCCA‐3′, and 5′‐GATCAAAGCCCTCGATGATC‐3′. β‐Arrestin2 knockout mice were backcrossed over seven generations to wild‐type control JAX™ C57BL/6J (RRID:IMSR_JAX:000664) mice from Charles River Laboratories (DE), which were also used for breeding of the knockout strain and as controls in all experiments. Mice were housed two to six per cage under a 12‐hr light–dark cycle (lights on at 6 a.m.) and ventilated at constant temperature of 21–23°C with ad libitum access to food and water. In all behavioural experiments, we used male mice aged 8–26 weeks and weighing 21–30 g. Studies were performed in parallel such that age‐matched mice received the same drug treatment at the same time. To avoid daytime effects, studies were carried out in small cohorts of mice (*n* = 6) at a time.

#### 2.1.2 Drugs

Drug doses were calculated according to the active component of the salt and were freshly prepared prior to use and diluted in PBS for injections. Morphine sulphate (3.75–52.5 mg·kg^−1^; Hameln Inc., Hameln, Germany) and fentanyl citrate (0.05–3 mg·kg^−1^; Rotexmedica, Trittau, Germany) were injected subcutaneously in lightly restrained, unanaesthetised mice at a volume of 10 μl·g^−1^ body weight.

#### 2.1.3 Mouse respiration experiments

In the respiration studies, one experimenter and a dedicated assistant performed all *in vivo* drug administrations and behavioural testing. Animals were assigned to groups randomly before testing. Mice were excluded from the study if they displayed any bodily injuries from aggressions with cage mates. The experimenter was blinded to treatment and/or genotype throughout the course of behavioural testing. All drugs were given to the experimenter in coded vials and decoded after completion of the experiment. All testing was conducted between 7 a.m. and 4 p.m. in an isolated, temperature‐ and light‐controlled room. Mice were acclimated to the facility for at least 2 weeks before testing. Only the experimenter and assistant had free access to the room and entered the room 30 min before commencement of testing to eliminate potential olfactory‐induced changes in nociception. All data from wild‐type and β‐arrestin2 knockout mice were generated in parallel with testing of phosphorylation‐deficient μ‐opioid receptor mice (S375A, 10S/T‐A and 11S/T‐A) published in Kliewer et al. (2019). Therefore, data from wild‐type mice shown in this paper were previously published. Respiratory rates were recorded with a nose‐out plethysmography system (Hugo Sachs Elektronik–Harvard Apparatus GmbH, DE). Individual unanaesthetised mice were placed in a restrainer with their nose exposed through a close‐fitting hole in the membrane. A pneumotachograph was connected to the chamber equipped with a differential low‐pressure transducer (transducer DLP2.5, Hugo Sachs Elektronik–Harvard Apparatus GmbH). Volume changes were calibrated by injecting known amounts of air into the chamber. Analogue pressure signals were digitised for later analysis (Levitt, Hunnicutt, Knopp, Williams, & Bissonnette, 2013). Mice were acclimated to the test chamber, and baseline respiratory parameters were recorded for 30 min. The 30‐min test was then repeated 15 min (fentanyl) or 30 min (morphine) after injection of different doses of fentanyl or morphine as indicated. Data were analysed in Pulmodyn® W Software (Hugo Sachs Elektronik–Harvard Apparatus GmbH).

#### 2.1.4 Accumulated faecal boli quantification

Mice were subcutaneously injected with vehicle or different doses of morphine or fentanyl as indicated and individually placed into small Plexiglas boxes (26.5 cm × 20.5 cm × 14 cm) lined with filter paper. Faecal boli were collected and weighed every hour for 3 hr (fentanyl) or 5 hr (morphine; Kliewer et al., 2019; Raehal et al., 2005).

### 2.2 Sydney, Australia

#### 2.2.1 Animals

β‐Arrestin2 knockout mice were kindly provided by Drs Lefkowitz and Caron (Duke University; Bohn, Gainetdinov, Lin, Lefkowitz, & Caron, 2000) and have been maintained as heterozygotes. Mice were genotyped by PCR analysis using the following primers: Forward 1 (5′‐TCTTCAAGAAGTCGAGCCCT‐3′), Forward 2 (5′‐GCTAAAGCGCATGCTCCAGA‐3′), and Reverse (5′‐ACAGGGTCCACTTTGTCCA‐3′). The knockout line was backcrossed with freshly purchased C57BL/6J wild‐type mice (The Animal Resources Centre, Perth, Western Australia) on at least four separate occasions since 2010 followed by extensive heterozygote X heterozygote mattings. β‐Arrestin2 knockout mice were offspring from a mix of heterozygote and homozygote crosses. Wild types were offspring from within the β‐arrestin2 knockout colony. Animals were housed no more than six per cage under a 12‐hr light–dark cycle (lights on at 6 a.m.) and ventilated at constant temperature of 21–23°C with ad libitum access to food and water. We used male mice between the ages of 6 and 18 weeks, with each experimental group comprising six to 11 mice per genotype per dose. Each animal was used for only a single dose.

#### 2.2.2 Drugs

Doses of morphine hydrochloride (3–100 mg·kg^−1^; GlaxoSmithKline, Australia) calculated as the active component of the salt were dissolved in saline and injected subcutaneously to lightly restrained, unanaesthetised mice at a maximum volume of 200 μl per mouse.

#### 2.2.3 Mouse respiration experiments

A single experimenter carried out all studies, and animals were randomly acclimatised to handling, the testing room and equipment before experimentation. Morphine‐induced changes to respiratory function were assessed on awake, freely moving mice using two parallel whole‐body plethysmography chambers connected to a pressure transducer with the accompanying controller and software (Buxco, DSI Instruments). A constant flow, calibrated before each experiment, of room air into each chamber is maintained by the system. Mice were pre‐acclimatised to the chamber for 20–25 min on the day before the experiment and were shielded from view of the other chamber. On the day of experimentation, animals were acclimatised for 25 min, of which the final 5 min were taken as a baseline. Mice were removed from the chamber for injection of morphine hydrochloride within a 2‐min window. Mice were returned to the chamber and respiratory function was assessed for the next 3 hr. Respiratory rate was determined by the system software (Buxco FinePointe, DSI Instruments) and averaged in 5‐min intervals. For time course analyses, parameters were normalised to the pre‐drug baseline as 100%. Peak drug effect on respiratory frequency was taken as the 5‐min period with the slowest breathing in the hour following each injection. Sleeping mice were excluded for that period. Data were reanalysed completely blind to the genotype by a separate experimenter familiar with the technique.

### 2.3 Bristol, United Kingdom

#### 2.3.1 Animals

Adult β‐arrestin2 knockout mice strain B6.129‐Arrb2^tm1Rjl^/J (βarr2−/−) and wild types from within the β‐arrestin2 knockout colony were kindly provided by Dr Stefan Schulz (Jena University, Germany). Animals were housed up to four per cage under a 12‐hr light–dark cycle (lights on at 8 a.m.) and ventilated at constant temperature of 21–23°C with ad libitum access to food and water. We used male mice weighing 21–30 g, with each experimental group comprising six mice per genotype. Studies were performed in parallel such that β‐arrestin2 knockout mice and wild‐type mice received the same drug treatment at the same time.

#### 2.3.2 Drugs

Doses of morphine hydrochloride (1–10 mg·kg^−1^; MacFarlan Smith, UK) calculated as weight of the salt were dissolved in saline and administrated intraperitoneally to lightly restrained, unanaesthetised mice in a volume of 0.1 ml. A single experimenter performed all *in vivo* drug administrations and respiration monitoring. Mice were assigned to groups randomly before testing. The experimenter was blinded to treatment and/or genotype throughout the course of the experiment. All drugs were given to the experimenter in coded vials and decoded after data analysis had been performed.

#### 2.3.3 Mouse respiration experiments

Experiments were conducted during the dark cycle between 8 a.m. and 8 p.m. using a red light. Mice were habituated to handling and the plethysmograph chambers prior to experimentation. Respiration was measured as previously described (Hill et al., 2016, 2018) in freely moving mice using whole‐body plethysmography chambers (EMKA Technologies, Paris, France) supplied with a 5% CO_2_ in air mixture (BOC Gas Supplies, Manchester, UK). Rate and volume of respiration were recorded and averaged over 5‐min periods. Breathing 5% CO_2_ in air increases min volume but does not induce stress in mice (Hill et al., 2016). Mice were habituated to plethysmograph chambers for 30 min on the day before the experiment. On the day of the experiment, baseline values of respiratory rate were recorded for 20 min, and then mice were removed from the chamber for 2 min for morphine injection after which respiration was measured for a further 40 min.

### 2.4 Animal experiments

The animal experiments conducted at Jena University Hospital were performed in accordance with the Thuringian state authorities, complied with the European Commission regulations for the care and use of laboratory animals, and were in accordance with the National Institutes of Health Guide for the Care and Use of Laboratory Animals. Those conducted at University of Sydney were performed under the guidelines of the Australian code of practice for the care and use of animals for scientific purposes (National Health and Medical Research Council, Australia, 7th Edition) and were approved by the University of Sydney Animal Ethics Committee. Those performed at the University of Bristol were conducted in accordance with the UK Animals (Scientific Procedures) Act 1986 and the European Communities Council Directive (2010/63/EU) and were approved by the University of Bristol Animal Welfare and Ethics Review Board. Animal studies are reported in compliance with the ARRIVE guidelines (Kilkenny, Browne, Cuthill, Emerson, & Altman, 2010) and with the recommendations made by the *British Journal of Pharmacology.*

### 2.5 Western blot analysis

Mice were anesthetised with isoflurane and killed by cervical dislocation, and brains were quickly dissected excluding the cerebellum. The remaining brain samples were immediately frozen in liquid nitrogen. Brains were transferred to ice‐cold detergent buffer (50‐mM Tris–HCl, pH 7.4, 150‐mM NaCl, 5‐mM EDTA, 10‐mM NaF, 10‐mM disodium pyrophosphate, 1% Nonidet P‐40, 0.5% sodium deoxycholate, 0.1% SDS, containing protease and phosphatase inhibitors), homogenised, and centrifuged at 14,000× *g* for 30 min at 4°C. The supernatant was added with SDS sample buffer, incubated for 5 min at 95°C, and then resolved on 8% SDS‐polyacrylamide gels. After electroblotting, membranes were incubated with the anti‐β‐arrestin2 (#3857, Cell Signaling, RRID:AB_2258681), anti‐β‐arrestin1 (ab32099, abcam, RRID:AB_722896) antisera followed by detection using an enhanced chemiluminescence detection system (Amersham, Braunschweig, Germany). Blots were subsequently stripped and reprobed with anti‐actin (sc‐47778, Santa Cruz Biotechnology, RRID:AB_626632) to confirm equal loading of the gels. Protein bands on western blots were exposed to X‐ray film. Films exposed in the linear range were then densitised using ImageJ 1.37v (RRID:SCR_003070). The Immuno‐related procedures used comply with the recommendations made by the *British Journal of Pharmacology* (Alexander et al., 2018).

### 2.6 Experimental design and data analysis

Data from previous experiments where respiratory depression or antinociception was measured following acute opioid administration in naïve mice were subjected to post hoc power analyses using G*Power (version 3.1.9, RRID:SCR_013726). Our calculations indicated that in acute respiration experiments, *n* = 6 for each individual group would produce a significant result if an actual effect occurred.

The results for each experiment were expressed as the means ± SEM. In the experiments performed in Germany, a cut‐off for respiratory rate was set at 60 and 360 breaths·min^−1^ for baseline measurement and 40 and 270 breaths·min^−1^ after morphine or fentanyl treatment.

Respiratory time course data from the United Kingdom and Australia were normalised to the pre‐drug baseline as 100%. Data are presented as percentage change from the pre‐drug baseline, calculated for each mouse individually before mean data were plotted. Presenting data as percentage change from the pre‐drug levels has been done to control for variation between treatment groups that may have different baseline levels of respiration. Normal distribution of the data was verified before performing parametric statistical analysis. Wherever appropriate, data were analysed using one‐way or two‐way ANOVA, followed by Bonferroni's post hoc tests. Statistical significance is assumed when *P* <0.05. All calculations were performed using GraphPad Prism 5, 6, or 7 software (GraphPad Software, Inc., San Diego, CA, RRID:SCR_002798). The data and statistical analysis comply with the recommendations of the *British Journal of Pharmacology* on experimental design and analysis in pharmacology (Curtis et al., 2015).

### 2.7 Data availability

The authors declare that all data supporting the findings of this study are presented within the paper and its supporting information files. The data that support the findings of this study are available from the authors upon reasonable request.

### 2.8 Nomenclature of targets and ligands

Key protein targets and ligands in this article are hyperlinked to corresponding entries in http://www.guidetopharmacology.org, the common portal for data from the IUPHAR/BPS Guide to PHARMACOLOGY (Harding et al., 2018), and are permanently archived in the Concise Guide to PHARMACOLOGY 2019/20 (Alexander et al., 2019).

## 3 RESULTS

**Figure 1:**
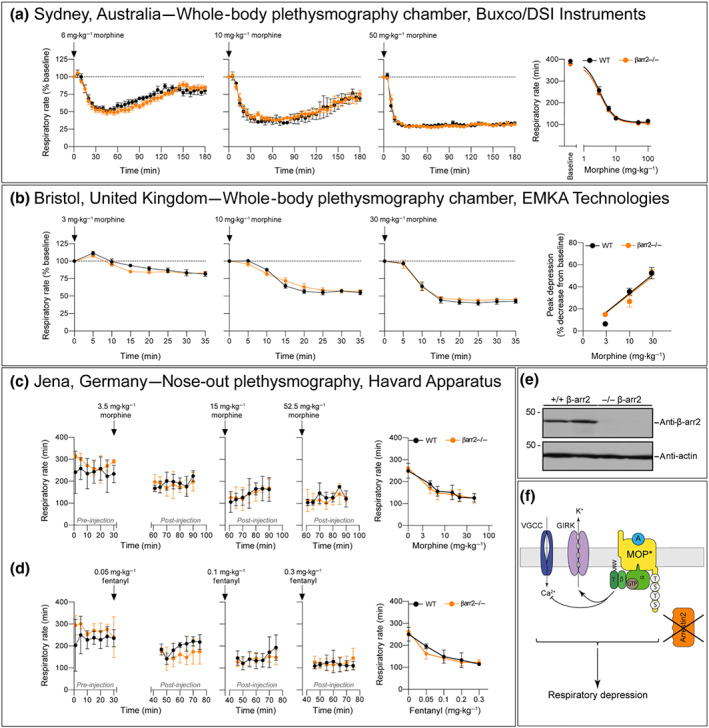
Respiratory depressant effect of morphine in β‐arrestin2 knockout mice. (a) Data: Sydney, Australia. Time course of respiratory rate depression following injection of 6, 10, or 50 mg·kg^−1^ of morphine s.c. measured with whole‐body plethysmography chambers (Buxco/DSI Instruments; *n* = 6–11). Parameters were normalised to the pre‐drug baseline as 100%. For dose–response curves, maximum depression of respiratory rate data from experiments in which mice were injected with 3–100 mg·kg^−1^ were fitted to a logistic function. (b) Data: Bristol, United Kingdom. Depression of respiratory rate following injection of 3, 10, and 30 mg·kg^−1^ morphine i.p. injection presented as respiratory rate (*n* = 6) measured with whole‐body plethysmography chambers (EMKA Technologies). The percentage respiratory rate following morphine injection for each animal was normalised to the pre‐drug baseline as 100%; baseline respiration rates before morphine injection in mice breathing 5% CO_2_ in air were 472.2 ± 23.7 and 484.5 ± 16.1 in wild‐type (WT) and β‐arrestin2 knockout mice, respectively (*n* = 6). Data are the means ± SEM. (c) Data: Jena, Germany. Time course of respiratory rate measured with a nose‐out plethysmography system (Harvard Apparatus) 30 min after 3.5, 15, or 52.5 mg·kg^−1^ of morphine s.c. (*n* = 6). Furthermore, dose–response curves of mean respiratory suppression over 30 min in which mice were injected with 3.5–52.5 mg·kg^−1^ are reported as the means ± SEM; baseline were set as 0. In (a)–(c), there was no statistical difference between morphine respiratory depression in β‐arrestin2 knockout and WT mice as determined by two‐way ANOVA with Bonferroni post hoc test. (d) Data: Jena, Germany. Time course of respiratory rate in WT and β‐arrestin2 knockout mice measured with nose‐out plethysmography system 15 min after 0.05, 0.1 or 0.3 mg·kg^−1^ of fentanyl s.c. (*n* = 6). Dose–response curves of respiratory suppression over 30 min in which mice were injected with 0.05–0.3 mg·kg^−1^ are reported as the means ± SEM; baselines were set as 0. Two‐way ANOVA with Bonferroni post hoc test. (e) Brain lysates from WT and β‐arrestin2 knockout mice (*n* = 2) were analysed for expression of β‐arrestin2. Blots were stripped and probed with anti‐actin antibody to confirm equal loading. The positions of molecular mass markers are indicated on the left (in kDa). (f) Schematic drawing of intracellular signalling cascades postulating that opioid‐induced respiratory depression is mediated by G protein signalling

In our studies, we utilised the same β‐arrestin2 knockout mouse line that was used in the initial study (Raehal et al., 2005). We backcrossed our β‐arrestin2 knockout mice over four to seven generations to wild‐type control C57BL/6J mice. The absence of β‐arrestin2 protein expression was confirmed for the mice used in each laboratory by western blotting and genotyping (Figures 1e and S2). We also confirmed that β‐arrestin1 protein expression is unchanged in brains of β‐arrestin2 knockout mice (Figure S1). Whole‐body plethysmography (Buxco/DSI Instruments) performed in Sydney, Australia, clearly showed a dose‐dependent depression of respiratory rate by morphine (6–100 mg·kg^−1^ s.c.) in both β‐arrestin2 knockout and wild‐type mice which persisted for over 3 hr following drug injection (Figure 1a). The predominant effect of morphine on respiration in mice is a depression of rate rather than depth of breathing (Hill et al., 2016). Similar results were obtained with a nose‐out plethysmography system (Harvard Apparatus) in Jena, Germany (Figure 1c). Respiratory rate was significantly reduced in both β‐arrestin2‐deficient and wild‐type mice after administration of morphine (3.75–52.5 mg·kg^−1^ s.c.; Figure 1c). Whole‐body plethysmography (EMKA Technologies) studies performed in Bristol, UK, also observed an equal, dose‐dependent depression of respiration by morphine (3–30 mg·kg^−1^ i.p.; Figure 1b) in both β‐arrestin2 knockout and wild‐type mice. Thus, three independent studies of respiratory depression by morphine using different plethysmography systems and data evaluations on independently bred transgenic mice have failed to find evidence that β‐arrestin2 signalling is involved in opioid‐induced respiratory depression (Figure 1f).

Fentanyl is a potent μ‐opioid agonist responsible for a large number of overdose deaths amongst drug users in North America (Lyden & Binswanger, 2019). The group which first reported that morphine did not induce respiratory depression in β‐arrestin2 knockout mice have recently suggested that fentanyl is β‐arrestin signalling biased and that this explains its propensity for causing severe respiratory depression (Schmid et al., 2017). We (the Jena group) therefore examined the ability of fentanyl to depress respiration in β‐arrestin2 knockout mice. Fentanyl (0.05–0.35 mg·kg^−1^ s.c.) produced a comparable depression of respiratory rate in both wild‐type and β‐arrestin2 knockout mice (Figure 1d).

**Figure 2:**
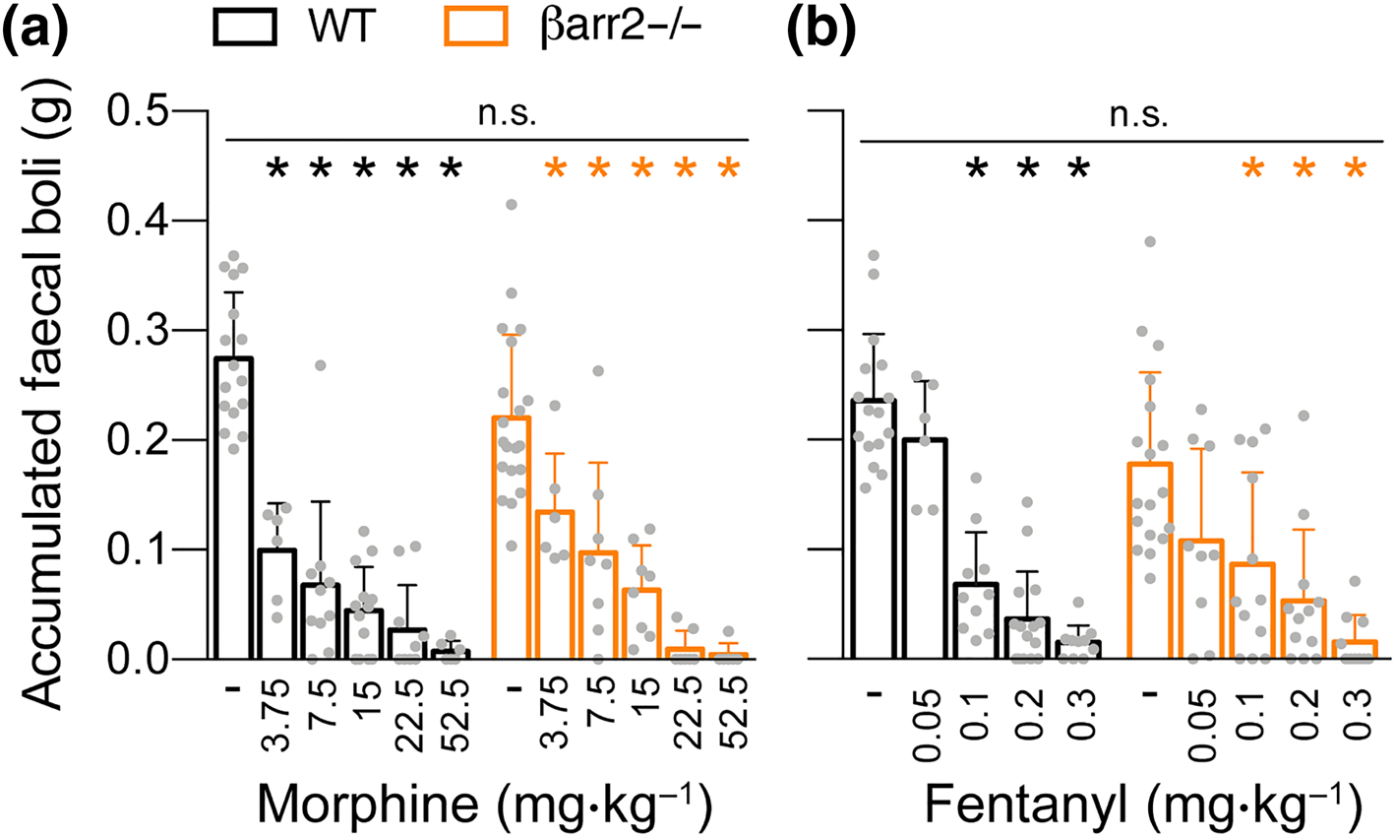
Opioid‐induced constipation in β‐arrestin2 knockout mice. Dose–response curves for accumulated faecal boli weight in the constipation test after (a) morphine and (b) fentanyl administration (*n* = 6–18). Data are the means ± SEM; *indicate statistically significant differences between drug and vehicle; n.s. indicates no statistically significant differences between genotypes; one‐way ANOVA with Bonferroni post hoc test. Data from Jena, Germany

Like respiratory depression, constipation is another classical μ‐opioid receptor‐mediated side effect. Quantification of accumulated faecal boli over a broad dose ranges of morphine and fentanyl showed profound constipation without significant difference between genotypes (Figure 2).

## 4 DISCUSSION

Biased agonism has been proposed as a means to separate desirable from adverse drug responses downstream of a GPCR target. However, for the μ‐opioid receptor, the biased agonism concept has two major weaknesses. First, it is predicated on a single study on β‐arrestin2 knockout mice that has never been reproduced in another laboratory. Second, the molecular pathways leading from β‐arrestin2 recruitment to respiratory depression *in vivo* have not been delineated. The finding of substantially reduced morphine‐induced respiratory depression in the β‐arrestin2 knockout mouse, reported by Raehal et al. (2005), is a central pillar of the notion that G protein bias drives opioid safety. However, the present study as tested in multiple laboratories independently failed to reproduce this phenotype.

At this time, we are unable to explain why our results contradict those originally published. The publication by Raehal et al. (2005) did not specify the background of mice or number of generations of inbreeding or back‐crossing that had been performed at that stage. The original study reporting the generation of the β‐arrestin2 knockout mouse and a phenotype of increased sensitivity to morphine antinociception was performed on a mixed C57BL/6 and 129SvJ background. Genetic analysis performed by the The Jackson Laboratory suggested that these mice may have been backcrossed to C57BL/6 for fewer than the reported 10 generations prior to arrival (https://www.jax.org/strain/011130). In fact, Raehal et al. (2005) acknowledged in their Methods that they had in some experiments used first‐generation offspring. Thus, we may speculate that the initial study was performed in part on a mixed C57BL/6 and 129SvJ background, which may provide one possible explanation for the differences between our study and the initial study. Indeed, at the recent 2019 International Narcotics Research Conference, it was reported by another group, led by Jennifer Whistler, that they too could not reproduce opioid respiratory depression in β‐arrestin2 knockout mice and also that 129SvJ mice showed little respiratory depression in response to morphine (Whistler, personal communication).

There is evidence for μ‐opioid receptor‐mediated respiratory depression being the result of G protein signalling through G_i_/G_o_ protein‐activated inwardly rectifying potassium channel (GIRK) activation. μ‐Opioid receptor activation by DAMGO or fentanyl leads to inhibition of rhythmic breathing (Montandon et al., 2016). Mice lacking the GIRK2 protein, an integral subunit of neuronal GIRK channels, did not show respiratory depression after systemic fentanyl or local DAMGO administered in the ventrolateral medulla. Furthermore, in wild‐type mice, direct inhibition of GIRK channels by local administration of Tertiapin‐Q markedly reduced DAMGO depression of respiration. Additional evidence showed that opioid‐induced, GIRK‐mediated hyperpolarisation of neurons in a distinct respiratory centre reduced respiratory rate (Kliewer et al., 2019; Levitt, Abdala, Paton, Bissonnette, & Williams, 2015). These results indicate that μ‐opioid receptor G_i_/G_o_ signalling through GIRK channels plays a pivotal role in opioid‐induced respiratory depression. The role of μ‐opioid receptor G_i_/G_o_ signalling through calcium channel inhibition has not been investigated to date.

Schmid et al. (2017) have proposed that fentanyl's ability to depress respiration results from it being an “arrestin‐biased” μ‐opioid receptor agonist (i.e. it is better at recruiting and signalling through arrestin than activating G protein signalling) and that opioid depression of respiration is mediated by arrestin signalling as proposed by Raehal et al. (2005). There are however two problems with their assertion. First, close inspection of their data reveals that fentanyl only showed arrestin bias when comparing arrestin recruitment with GTPyS binding. Fentanyl did not exhibit bias when comparing arrestin recruitment with inhibition of cAMP accumulation despite μ‐opioid receptor inhibition of cAMP accumulation being G protein mediated. Furthermore, we have previously reported that in our hands fentanyl does not exhibit arrestin bias in arrestin translocation and GTPyS binding assays (McPherson et al., 2010; Rivero et al., 2012). Apparent bias may be a consequence of the experimental protocol and *in vitro* assay system utilised (Thompson et al., 2016). Thus, care should be taken when extrapolating *in vitro* profiles to link a specific receptor signalling process to a behavioural response.

Second, in transgenic mice in which all the phosphorylation sites on the C‐tail of the μ‐opioid receptor had been mutated to alanine, fentanyl still depressed respiration (Kliewer et al., 2019). In these transgenic mice, μ‐opioid receptors are not phosphorylated by G protein receptor kinases and thus do not bind arrestins. This observation brings into question the concept that opioid depression of respiration is a function of arrestin signalling (Montandon & Slutsky, 2019). We have reported that PZM21, a purportedly G protein‐biased ligand, depresses respiration in mice (Hill et al., 2018) despite an initial report to the contrary (Manglik et al., 2016).

Together, this collaborative study unequivocally demonstrates the persistence of opioid‐induced respiratory depression in β‐arrestin2 knockout mice. This is in line with our recently reported finding that knock‐in of phosphorylation‐deficient μ‐opioid receptors that cannot recruit arrestins does not improve opioid safety (Kliewer et al., 2019). Both sets of results bring into question the concept of developing drugs that activate G proteins better than they recruit arrestins (i.e. “G protein‐biased agonists”), as a strategy for the development of safer opioid analgesic drugs.

## ACKNOWLEDGEMENTS

This work was supported by the Deutsche Forschungsgemeinschaft Grants SFB/TR166‐TPC5, SCHU924/11‐3, and SCHU924/18‐1 to S.S., National Health and Medical Research Council of Australia (APP1072113 and 1045964) to M.J.C., and National Institute of Health (USA) Grant RO1DA036975 to G.H.

## AUTHOR CONTRIBUTIONS

E.K., G.H., M.J.C., and S.S. initiated the project and designed all behavioural pharmacology experiments with A.K., A.G., and R.H. A.G., R.H., A.K., and F.S. performed mouse plethysmography studies. The manuscript was written by S.S., A.K., E.K., G.H., M.J.C., A.G., R.H., and C.B.

## CONFLICT OF INTEREST

The authors declare no conflicts of interest.

## DECLARATION OF TRANSPARENCY AND SCIENTIFIC RIGOUR

This Declaration acknowledges that this paper adheres to the principles for transparent reporting and scientific rigour of preclinical research as stated in the *BJP* guidelines for Design & Analysis, Immunoblotting and Immunochemistry, and Animal Experimentation, and as recommended by funding agencies, publishers, and other organisations engaged with supporting research.

## Supporting information

Figure S1. Western blot analysis of β‐arrestin2 knockout mice. Brain lysates from WT and β‐arrestin2 knockout mice (*n* = 3) were analysed for expression of βarrestin1. Blots were stripped and probed with anti‐actin antibody to confirm equal loading. The positions of molecular mass markers are indicated on the left (in kDa)
Figure S2. PCR analysis of β‐arrestin2 knsockout mice. A. Data: Jena, Germany. Mice were genotyped by PCR analysis of genomic tail‐biopsy DNA using the following primers: Forward 5′‐GCTAAAGCGCATGCTCCAGA‐3′, Reverse 5′‐ACAGGGTCCACTTTGTCCA‐3′ and 5′‐GATCAAAGCCCTCGATGATC‐3′. B. Data: Sydney, Australia. Mice were genotyped by PCR analysis using the following primers: Forward 1 5′‐TCTTCAAGAAGTCGAGCCCT‐3′, Forward 2 5′‐GCTAAAGCGCATGCTCCAGA‐3′, and Reverse 5′‐ACAGGGTCCACTTTGTCCA‐3′

## References

[bph15004-bib-0001] Alexander, S. P. H. , Christopoulos, A. , Davenport, A. P. , Kelly, E. , Mathie, A. , Peter, J. A. , … CGTP Collaborators (2019). The concise guide to pharmacology 2019/2020: G protein‐coupled receptors. British Journal of Pharmacology, 176, S21–S141. 10.1111/bph.14748 31710717 PMC6844580

[bph15004-bib-0002] Alexander, S. P. H. , Roberts, R. E. , Broughton, B. R. S. , Sobey, C. G. , George, C. H. , & Stanford, S. C. (2018). Goals and practicalities of immunoblotting and immunohistochemistry: A guide for submission to the British Journal of Pharmacology. British Journal of Pharmacology, 175, 407–411. 29350411 10.1111/bph.14112 PMC5773976

[bph15004-bib-0003] Bohn, L. M. , Gainetdinov, R. R. , Lin, F. T. , Lefkowitz, R. J. , & Caron, M. G. (2000). μ‐Opioid receptor desensitization by β‐arrestin‐2 determines morphine tolerance but not dependence. Nature, 408, 720–723. 10.1038/35047086 11130073

[bph15004-bib-0004] Curtis, M. J. , Bond, R. A. , Spina, D. , Ahluwalia, A. , Alexander, S. P. , Giembycz, M. A. , … McGrath, J. (2015). Experimental design and analysis and their reporting: New guidance for publication in BJP. British Journal of Pharmacology, 172, 3461–3471. 10.1111/bph.12856 26114403 PMC4507152

[bph15004-bib-0005] Harding, S. D. , Sharman, J. L. , Faccenda, E. , Southan, C. , Pawson, A. J. , Ireland, S. , … NC‐IUPHAR (2018). The IUPHAR/BPS Guide to PHARMACOLOGY in 2018: Updates and expansion to encompass the new guide to IMMUNOPHARMACOLOGY. Nucleic Acids Research, 46, D1091–D1106. 10.1093/nar/gkx1121 29149325 PMC5753190

[bph15004-bib-0006] Hertz, S. (2018). FDA briefing document overview of the October 11, 2018 AADPAC meeting to discuss NDA 210730. https://www.fda.gov/media/121233/download .

[bph15004-bib-0007] Hill, R. , Disney, A. , Conibear, A. , Sutcliffe, K. , Dewey, W. , Husbands, S. , … Henderson, G. (2018). The novel μ‐opioid receptor agonist PZM21 depresses respiration and induces tolerance to antinociception. British Journal of Pharmacology, 175, 2653–2661. 10.1111/bph.14224 29582414 PMC6003631

[bph15004-bib-0008] Hill, R. , Lyndon, A. , Withey, S. , Roberts, J. , Kershaw, Y. , MacLachlan, J. , … Henderson, G. (2016). Ethanol reversal of tolerance to the respiratory depressant effects of morphine. Neuropsychopharmacology, 41, 762–773. 10.1038/npp.2015.201 26171718 PMC4610039

[bph15004-bib-0009] Kilkenny, C. , Browne, W. , Cuthill, I. C. , Emerson, M. , & Altman, D. G. (2010). Animal research: Reporting in vivo experiments: The ARRIVE guidelines. British Journal of Pharmacology, 160, 1577–1579. 20649561 10.1111/j.1476-5381.2010.00872.x PMC2936830

[bph15004-bib-0010] Kliewer, A. , Schmiedel, F. , Sianati, S. , Bailey, A. , Bateman, J. T. , Levitt, E. S. , … Schulz, S. (2019). Phosphorylation‐deficient G‐protein‐biased μ‐opioid receptors improve analgesia and diminish tolerance but worsen opioid side effects. Nature Communications, 10, 367 10.1038/s41467-018-08162-1 PMC6341117 30664663

[bph15004-bib-0011] Levitt, E. S. , Abdala, A. P. , Paton, J. F. , Bissonnette, J. M. , & Williams, J. T. (2015). μ‐Opioid receptor activation hyperpolarizes respiratory‐controlling Kölliker–Fuse neurons and suppresses post‐inspiratory drive. The Journal of Physiology, 593, 4453–4469. 10.1113/JP270822 26175072 PMC4594241

[bph15004-bib-0012] Levitt, E. S. , Hunnicutt, B. J. , Knopp, S. J. , Williams, J. T. , & Bissonnette, J. M. (2013). A selective 5‐HT_1a_ receptor agonist improves respiration in a mouse model of Rett syndrome. Journal of Applied Physiology, 115, 1626–1633. 10.1152/japplphysiol.00889.2013 24092697 PMC3882741

[bph15004-bib-0013] Lyden, J. , & Binswanger, I. A. (2019). The United States opioid epidemic. Seminars in Perinatology, 43, 123–131. 10.1053/j.semperi.2019.01.001 30711195 PMC6578581

[bph15004-bib-0014] Manglik, A. , Lin, H. , Aryal, D. K. , McCorvy, J. D. , Dengler, D. , Corder, G. , … Shoichet, B. K. (2016). Structure‐based discovery of opioid analgesics with reduced side effects. Nature, 537, 185–190. 10.1038/nature19112 27533032 PMC5161585

[bph15004-bib-0015] McPherson, J. , Rivero, G. , Baptist, M. , Llorente, J. , Al‐Sabah, S. , Krasel, C. , … Henderson, G. (2010). μ‐Opioid receptors: Correlation of agonist efficacy for signalling with ability to activate internalization. Molecular Pharmacology, 78, 756–766. 10.1124/mol.110.066613 20647394 PMC2981392

[bph15004-bib-0023] Montandon, G. , & Slutsky, A. S. (2019). Solving the opioid crisis: respiratory depression by opioids as critical end point. Chest, 156(4), 653–658. 31194974 10.1016/j.chest.2019.05.015

[bph15004-bib-0016] Montandon, G. , Ren, J. , Victoria, N. C. , Liu, H. , Wickman, K. , Greer, J. J. , & Horner, R. L. (2016). G‐protein‐gated inwardly rectifying potassium channels modulate respiratory depression by opioids. Anesthesiology, 124, 641–150. 10.1097/ALN.0000000000000984 PMC4755838 26675532

[bph15004-bib-0017] Raehal, K. M. , Walker, J. K. , & Bohn, L. M. (2005). Morphine side effects in β‐arrestin 2 knockout mice. The Journal of Pharmacology and Experimental Therapeutics, 314, 1195–1201. 10.1124/jpet.105.087254 15917400

[bph15004-bib-0018] Reiter, E. , Ahn, S. , Shukla, A. K. , & Lefkowitz, R. J. (2012). Molecular mechanism of β‐arrestin‐biased agonism at seven‐transmembrane receptors. Annual Review of Pharmacology and Toxicology, 52, 179–197. 10.1146/annurev.pharmtox.010909.105800 PMC3628752 21942629

[bph15004-bib-0019] Rivero, G. , Llorente, J. , McPherson, J. , Cooke, A. , Mundell, S. J. , McArdle, C. A. , … Kelly, E. (2012). Endomorphin‐2: A biased agonist at the μ‐opioid receptor. Molecular Pharmacology, 82, 178–188. 10.1124/mol.112.078659 22553358 PMC3400840

[bph15004-bib-0020] Schmid, C. L. , Kennedy, N. M. , Ross, N. C. , Lovell, K. M. , Yue, Z. , Morgenweck, J. , … Bohn, L. M. (2017). Bias factor and therapeutic window correlate to predict safer opioid analgesics. Cell, 171(1165–1175), e1113. 10.1016/j.cell.2017.10.035 PMC5731250 29149605

[bph15004-bib-0021] Thompson, G. L. , Lane, J. R. , Coudrat, T. , Sexton, P. M. , Christopoulos, A. , & Canals, M. (2016). Systematic analysis of factors influencing observations of biased agonism at the mu‐opioid receptor. Biochemical Pharmacology, 113, 70–87. 10.1016/j.bcp.2016.05.014 27286929

[bph15004-bib-0022] Wacker, D. , Stevens, R. C. , & Roth, B. L. (2017). How ligands illuminate GPCR molecular pharmacology. Cell, 170, 414–427. 10.1016/j.cell.2017.07.009 28753422 PMC5560499

