# Supplementary material for: Morphine‐induced respiratory depression is independent of β‐arrestin2 signalling
Source: Br J Pharmacol. 2020 Feb 17;177(13):2923–31. doi: 10.1111/bph.15004 (PMC7280004; doi:10.1111/bph.15004)
Supplement: Supplementary file 1 — Figure S1. Western blot analysis of β‐arrestin2 knockout mice. Brain lysates from WT and β‐arrestin2 knockout mice (n = 3) were analysed for expression of βarrestin1. Blots were stripped and probed with anti‐actin antibody to confirm equal loading. The positions of molecular mass markers are indicated on the left (in kDa) Figure S2. PCR analysis of β‐arrestin2 knsockout mice. A. Data: Jena, Germany. Mice were genotyped by PCR analysis of genomic tail‐biopsy DNA using the following primers: Forward 5′‐GCTAAAGCGCATGCTCCAGA‐3′, Reverse 5′‐ACAGGGTCCACTTTGTCCA‐3′ and 5′‐GATCAAAGCCCTCGATGATC‐3′. B. Data: Sydney, Australia. Mice were genotyped by PCR analysis using the following primers: Forward 1 5′‐TCTTCAAGAAGTCGAGCCCT‐3′, Forward 2 5′‐GCTAAAGCGCATGCTCCAGA‐3′, and Reverse 5′‐ACAGGGTCCACTTTGTCCA‐3′ [file BPH-177-2923-s001.pdf]

Morphine-induced respiratory depression is independent of ß-arrestin2 signalling

Andrea Kliewer[1*], Alexander Gillis[2*], Rob Hill[3*], Frank Schmidel[1], Chris Bailey[4], Eamonn Kelly[3#], Graeme Henderson[3#], Macdonald J Christie[2#] and Stefan Schulz[1#]

[1]Institute of Pharmacology and Toxicology, Jena University Hospital, Friedrich-Schiller-University, 07747 Jena, Germany

[2]Discipline of Pharmacology, School of Medical Sciences, University of Sydney, NSW 2006, Australia.

[3]School of Physiology, Pharmacology and Neuroscience, University of Bristol, Bristol, UK

[4]Department of Pharmacy and Pharmacology, University of Bath, Bath, UK

*contributed equally as first author

#contributed equally as last author

Running Head: Opioid-induced respiratory depression in ß-arrestin2 KO mice

Authors for correspondence: E.K. (E.Kelly@bristol.ac.uk), G.H. (Graeme.Henderson@bristol.ac.uk), M.J.C. (mac.christie@sydney.edu.au) and S.S. (stefan.schulz@med.uni-jena.de).

Financial disclosures: None

Statement of Conflicts of Interest: The authors have nothing to disclose

Supplementary Figure 1

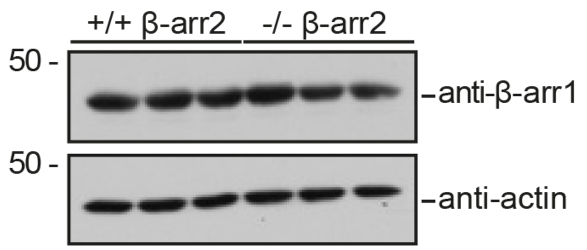

Supplementary Figure 1. Western blot analysis of ß-arrestin2 knockout mice. Brain lysates from WT and ß-arrestin2 knockout mice (n = 3) were analysed for expression of ß-arrestin1. Blots were stripped and probed with anti-actin antibody to confirm equal loading. The positions of molecular mass markers are indicated on the *left* (in kDa).

Supplementary Figure 2

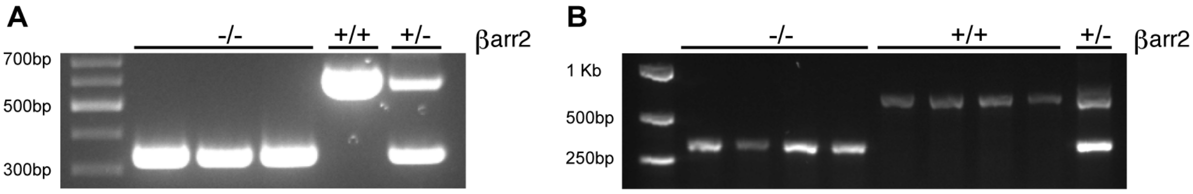

Supplementary Figure 2. PCR analysis of ß-arrestin2 knockout mice. A. *Data: Jena, Germany.* Mice were genotyped by PCR analysis of genomic tail-biopsy DNA using the following primers: Forward 5′-GCTAAAGCGCATGCTCCAGA-3′, Reverse 5′-ACAGGGTCCACTTTGTCCA-3′ and 5′-GATCAAAGCCCTCGATGATC-3′. **B.** *Data: Sydney, Australia*. Mice were genotyped by PCR analysis using the following primers: Forward 1 5'-TCTTCAAGAAGTCGAGCCCT-3', Forward 2 5'-GCTAAAGCGCATGCTCCAGA-3', and Reverse 5'-ACAGGGTCCACTTTGTCCA-3'.
